# *Toxoplasma gondii* is not an important contributor to poor reproductive performance of primiparous ewes from southern Australia: a prospective cohort study

**DOI:** 10.1186/s12917-022-03211-w

**Published:** 2022-03-19

**Authors:** Thomas Clune, Amy Lockwood, Serina Hancock, Andrew N. Thompson, Mieghan Bruce, Sue Beetson, Angus J. Campbell, Elsa Glanville, Daniel Brookes, Colin Trengove, Ryan O’Handley, Caroline Jacobson

**Affiliations:** 1 Centre for Animal Production and Health, Food Futures Institute, Murdoch University, South Street, Murdoch, Western Australia 6150 Australia; 2 Nossal Institute for Global Health, Melbourne School of Population and Global Health, University of Melbourne, Melbourne, Victoria 3010 Australia; 3 Mackinnon Project, Faculty of Veterinary & Agricultural Sciences, University of Melbourne, Werribee, Victoria 3030 Australia; 4 School of Animal and Veterinary Science, University of Adelaide, Roseworthy, South Australia 5371 Australia

**Keywords:** Toxoplasmosis, Lamb survival, Abortion, Lamb mortality, Sheep, Parasite, Reproduction

## Abstract

**Background:**

*Toxoplasma gondii* causes reproductive losses in sheep worldwide, including Australia. The reproductive performance of primiparous ewes is typically lower than for mature, multiparous ewes, and younger ewes are more likely to be immunologically naïve and therefore more susceptible to reproductive disease if *T. gondii* infection occurs during pregnancy. The aim of this study was to assess the impact of infection with *T. gondii* on the reproductive performance of primiparous ewes in southern Australia using a prospective cohort study. This will inform the need for targeted control strategies for *T. gondii* in Australian sheep.

**Results:**

*Toxoplasma gondii* seropositivity using indirect ELISA was detected at 16/28 farms located across southern Australia. Apparent seropositivity to *T. gondii* was lower in primiparous ewes (1.1, 95% confidence interval (CI) 0.6, 1.8) compared to mature, multiparous ewes (8.1, 95% CI 6.0, 10.5; *P* < 0.001). *Toxoplasma gondii* seroconversion during the gestation and lambing period was confirmed for 11/1097 (1.0, 95% CI 0.5, 1.7) of pregnant primiparous ewes that failed to raise a lamb, and 1/161 (0.6, 95% CI 0.1, 2.9) primiparous ewes with confirmed mid-pregnancy abortion.

**Conclusions:**

Low frequency of detection of *T. gondii* seroconversion during gestation and low frequency of seropositivity to *T. gondii* suggests that toxoplasmosis was not an important contributor to reproductive losses in primiparous ewes on farms located over a wide geographical area in southern Australia.

**Supplementary Information:**

The online version contains supplementary material available at 10.1186/s12917-022-03211-w.

## Background

The number of lambs weaned from primiparous ewes is typically lower and more variable compared to multiparous ewes [1, 2]. However, little research has investigated the causes of foetal and lamb mortality occurring between pregnancy diagnosis in mid-pregnancy and weaning for primiparous ewes. Toxoplasmosis is a globally important disease of sheep that is caused by infection with *Toxoplasma gondii* and can cause early embryonic deaths, abortions, stillbirths, premature lambs and the birth of weak lambs that have poor survival rates. Zoonotic transmission of *T. gondii* via ingestion of viable cysts in undercooked sheepmeat is also an important public health issue [3].

Sheep can be infected with *T. gondii* via ingestion of feed or water contaminated with oocysts that have been shed by a feline definitive host [4]. Vertical transmission after pregnancy-induced recrudescence of persistent infections has also been reported [5, 6]. Reproductive disease is generally observed only following a primary infection in a naïve pregnant ewe, and *T. gondii* infection usually confers long-lasting protective immunity [7]. Hence, the likelihood of infection and thus immunity increases with age [8]. Young ewes are therefore more likely to be immunologically naïve and susceptible to reproductive disease if exposed to infective oocysts during gestation.

Toxoplasmosis is one of the most commonly diagnosed causes of abortion in ewes in southern Australia [9]. However, the incidence of reproductive disease associated with toxoplasmosis in Australian ewes is not well described. Serological surveys conducted in Australian sheep have demonstrated that *T. gondii* has a broad geographical distribution with reports of seropositivity on 41–97% of the studied farms and mean individual animal seropositivity ranging from 7 to 62% (Additional file 1). However, most of these studies were restricted to specific regions or do not discriminate between age groups of sheep.

Whilst *T. gondii* is endemic in Australia, its impact on ewe reproduction, and specifically reproduction for primiparous ewes, is not well quantified. A seroprevalence survey in South Australia reported a negative correlation between within-flock *T. gondii* seroprevalence and lamb marking rate [10]. However, this study did not investigate seroprevalence between ewe age groups or determine the timing of *T. gondii* seroconversion relative to reproductive outcome. Similarly, a study in Uruguay identified lower lambing rates for ewes that seroconverted for *T. gondii* during gestation [11]. Vaccination against *T. gondii* was associated with increased lamb marking percentages in extensively managed primiparous ewe lambs in New Zealand which suggests that toxoplasmosis was impacting reproduction in flocks that were not vaccinated [12].

The aim of this study was to assess the impact of *T. gondii* infection on the reproductive performance of primiparous ewes in southern Australia. This will inform the need for targeted control strategies for *T. gondii* in Australian sheep.

## Results

### Primiparous ewe reproductive performance

Foetal loss and lamb mortality for progeny of primiparous ewes have been reported in more detail by Clune et al. [13]. Briefly, foetal and lamb mortality between scan 1 and lamb marking was 36% (1567/4351 foetuses; range 14–71%) for primiparous ewe lambs and 28% (582/2103 foetuses; range 20–53%) for primiparous yearlings. Mid-pregnancy abortion was detected in 14/19 primiparous ewe lamb flocks and 6/11 primiparous yearling flocks. In primiparous ewe lamb flocks, mid-pregnancy abortion was detected in 5.2% (155/2968) ewes, ranging 0–50.0% across flocks. In primiparous yearling flocks, mid-pregnancy abortion was detected in 0.8% (16/1886) ewes, ranging 0–4.4% across flocks.

### *Toxoplasma gondii* seropositivity

Apparent and true *T. gondii* seroprevalence for ewe age categories are shown in Table 1. Apparent *T. gondii* seroprevalence for primiparous ewes (ewe lambs and yearlings combined) was 1.1% (95% CI 0.6, 1.8). Apparent individual-animal *T. gondii* seroprevalence was higher for mature ewes compared to primiparous ewe lambs (*P* < 0.001) and primiparous yearling ewes (*P* < 0.001). There was no difference in the apparent seroprevalence between primiparous ewes mated as ewe lambs or yearlings (*P* = 0.214).

**Table 1:** Apparent and estimated true seropositivity to *T. gondii* for primiparous ewes mated as ewe lambs (approximately one-year-old at sampling) or yearlings (approximately two-years-old at sampling) and mature multiparous ewes (aged three-years-old or older) from 28 Australian farms

	Ewes sampled				
	Flocks (*n*)	Individual ewes (*n*)	Seropositive samples (*n*)	Apparent seropositivity % (95% CI)	Estimated true seropositivity % (95% CrI)
**Primiparous ewes**					
Ewe lambs	19	839	7	0.8 (0.4, 1.6)^a^	0.7 (0.1, 1.6)
Yearling	11	440	7	1.6 (0.7, 3.1)^a^	1.6 (0.4, 3.1)
**Mature ewes**	28	558	45	8.1 (6.0, 10.5)^b^	8.1 (5.8, 10.6)

95% CI: 95% confidence interval

95% CrI: 95% credible interval

^ab^ Apparent seropositivity proportion (%) with different superscripts are significantly different (two sample proportion z-test (2-tailed) *P* < 0.05)

*Toxoplasma gondii* seropositivity was detected in at least one ewe for 16/28 (57%) of the farms. *Toxoplasma gondii* seropositivity was identified in 12/30 (40%) of primiparous ewe flocks and 11/28 (39%) of mature ewe flocks. For flocks where *T. gondii* seropositivity was detected, within-flock seroprevalence ranged from 1 to 5% for primiparous ewes (Additional File 2) and 5–50% for mature ewes (Additional File 3). The majority (82%) of seropositive mature ewes were detected on five farms where within-flock seroprevalence for mature ewes ranged 25–50% (Additional File 3).

There was no difference in the proportion of primiparous ewes seropositive to *T. gondii* between states (Table 2). The proportion of mature ewes that were seropositive to *T. gondii* was greater for South Australia and Victoria compared to Western Australia (Table 2).

**Table 2:** Apparent seropositivity to *T. gondii* at state-level for primiparous ewes mated as ewe lambs (approximately one-year-old at sampling) or yearlings (approximately two-years old at sampling) and mature multiparous ewes (aged 3 years or older) from 28 Australian farms

State^	Ewe lambs		Yearlings		Mature ewes	
	Sampled (*n*)	Seropositive (*n* (%))	Sampled (*n*)	Seropositive (*n* (%))	Sampled (*n*)	Seropositive (*n* (%))
WA	338	2 (0.6)^a^	160	3 (1.9)^a^	200	5 (2.5)^a^
SA	221	2 (0.9)^a^	160	2 (1.3)^a^	178	17 (9.6)^b^
VIC	280	3 (1.1)^a^	120	2 (1.7)^a^	180	23 (12.8)^b^

^SA: South Australia; WA: Western Australia; VIC: Victoria.

^ab^ Apparent seropositivity proportions (%) within columns with different superscripts are significantly different (two sample proportion z-test (two-tailed) *P* < 0.05)

### Timing of *T. gondii* seroconversion in primiparous ewes

A total of 1279 primiparous ewes were screened for *T. gondii* seropositivity (Table 1), of which 1097 were pregnant at scan 1 but failed to rear a lamb to marking (Additional file 2). Of the 1097 ewes that were tested and failed to rear a lamb, mid pregnancy abortion (pregnancy loss between scan 1 and scan 2) was detected in 161 ewes.

*Toxoplasma gondii* seropositivity was detected for 7 ewes joined as ewe lambs and 7 ewes joined as yearlings (Table 1). Of these, outcome of pregnancy and serial blood samples were available for 12 ewes. The timing of detection of seroconversion in these 12 ewes is shown in Table 3*.* Seropositivity to *T. gondii* at mating was detected in 1/12 ewes that were seropositive to *T. gondii* at lamb marking (or last available sample). Seroconversion to *T. gondii* after mating was detected for 11/1097 (1.0, 95% CI 0.5, 1.7) of the primiparous ewes that failed to raise a lamb based on detection of seropositivity at lamb marking (or last available sample) but not at mating.

**Table 3:** Timing of detection for *T. gondii* IgG seroconversion using indirect ELISA for primiparous ewes (*n* = 12) sampled across southern Australia between 2018 and 2020. The earliest detection of seroconversion is bolded

Ewe ID	Farm	Timing of foetal or lamb loss	***T. gondii*** seroconversion status				
			Pre-mating	Scan 1	Scan 2	Pre-lambing	Lamb marking
**Seroconversion detected pre-mating**							
9463	13	Perinatal death	**Positive**	Positive	Positive	NA	Positive
**Seroconversion first detected during pregnancy**							
18842	20	Late abortion/ perinatal lamb death	Negative	**Positive**	Positive	NA	Positive
18857	20	Perinatal death	Negative	**Positive**	Positive	NA	Positive
3479	3	Late abortion/ perinatal lamb death	Negative	**Positive**	Negative	Negative	Positive
12114	12	Mid Abortion (scan 2 – scan 3)	Negative	Negative	**Positive**	NA	NA
13091	7	Late abortion ^a^ (scan 3 – pre-lambing)	Negative	Negative	Negative	**Positive**	NA
18121	21	Perinatal death	Negative	Negative	Negative	**Positive**	Positive
**Seroconversion first detected at marking**							
16527	10	Perinatal death	Negative	Negative	Negative	NA	**Positive**
16528	10	Perinatal death	Negative	Negative	Negative	NA	**Positive**
17152	15	Perinatal death	Negative	Negative	Negative	Negative	**Positive**
18190	22	Perinatal death	Negative	Negative	Negative	Negative	**Positive**
23364	29	Raised twins	Negative	NA	NA	Negative	**Positive**

*NA* Not available for testing as the ewe was not present for sampling or had been removed from the study flock after abortion was confirmed

^a^confirmed late abortion based on observation of purulent vaginal discharge and opportunistic transabdominal ultrasound at the pre-laambing visit

For the subset of ewes selected for serology that had mid-pregnancy abortion, 1/161 (0.6, 95% CI 0.1, 2.9) ewes had evidence of *T. gondii* seroconversion after mating evident as *T. gondii* seropositivity at marking and with no evidence of seropositivity at mating (ewe ID 12114; Table 3). Seropositivity was also detected in a second ewe that aborted but did not have serial blood samples available to determine timing of seroconversion. One ewe that was opportunistically identified with late-pregnancy abortion had evidence of *T. gondii* seroconversion after mating (ewe ID 13091; Table 3).

Serum samples categorised as negative for *T. gondii* IgG at a sampling timepoint after detection of *T. gondii* seroconversion were detected for only one ewe (Ewe 3479; Table 3).

## Discussion

Toxoplasmosis was not an important contributor to foetal and lamb mortality between pregnancy scanning and marking for the primiparous ewe flocks in this study. Whilst there was serological evidence of widespread exposure to *T. gondii* at farm level, seroconversion after mating was evident for only 1% of primiparous ewes that were confirmed to be pregnant and subsequently failed to raise a lamb. Low frequency of *T. gondii* seropositivity in primiparous ewes was consistent with the absence of detection of *T. gondii* using qPCR on aborted and stillborn lambs from a subset of farms in this study previously reported by Clune et al. [14] and described in more detail in Additional File 4. These findings are in accord with a recent review of submissions to Australian veterinary laboratories that reported *T. gondii* was implicated in 5% of sheep abortion investigations, and suggests that toxoplasmosis is a sporadic cause of abortion in Australian sheep [9]. Our findings indicate that routine vaccination for toxoplasmosis is unlikely to be economically justified for many Australian sheep producers unless there is evidence demonstrating high risk of exposure to *T. gondii* in the specific region.

This study used serial serology to assess the timing of seroconversion relative to abortion or lamb death. Abortions may occur acutely, or up to 8 weeks post-infection, with the outcome of infection being largely dependent on the stage of gestation when *T. gondii* infection occurred [15, 16]. *Toxoplasma gondii* IgG antibodies are detectable by P30 ELISA between 3 and 10 weeks after infection [17] and persist for several years [18]. So, it is unlikely that antibodies would have failed to rise to detectable levels or waned to below detectable levels by lamb marking if ewes had become infected with *T. gondii* during pregnancy and foetal or lamb mortality was related to toxoplasmosis. Based on these assumptions, toxoplasmosis was a plausible aetiology for abortions and lamb mortalities in ewes 12114, 13091, 18842 and 18857 (Table 3). However, attempting to relate maternal serostatus to the occurrence of abortion or lamb mortality can be unreliable due to the variable timeframe between detection of seroconversion and lamb mortality [16]. Toxoplasmosis-associated reproductive disease may be confirmed using foetal serology [16], molecular diagnostics [19], and histopathology [20]. Additionally, quantitative serology to evaluate changes in antibody titres for ewes with suspected toxoplasmosis would allow more accurate interpretation of serial serological results. Nevertheless, even if foetal and lamb mortality for all ewes that were seropositive at lamb marking in this study were due to toxoplasmosis, this still represented a very small contributor to overall foetal and lamb mortality in these flocks.

The unclear association between timing of *T. gondii* seroconversion and abortion in some ewes was also consistent with observations that other factors were likely to be contributing to abortion and perinatal lamb deaths in these flocks. Abortions, stillbirths and polyarthritis associated with *Chlamydia pecorum* were detected in primiparous ewe flocks from Western Australia, and non-infectious causes of death (including dystocia and starvation-mismothering) were important contributors to cause of lamb death identified at necropsy [14, 21]. Previous Australian studies have reported dystocia and starvation-mismothering to be important contributors to perinatal lamb mortality [22–24], and sporadic abortion associated with *C. pecorum* infections in ewe lambs [25]. There was no evidence neosporosis or coxiellosis were contributing to foetal or lamb mortality in the same flocks reported in this study [26, 27]. Abortions associated with *Campylobacter fetus fetus* were detected by microbial culture from aborted lambs in one flock included in this study [28]. A review of abortion investigations submitted to Australian veterinary laboratories reported campylobacteriosis and listeriosis to be the most common diagnoses made for investigations of ovine abortion or stillbirth [9].

This study reported seropositivity for ewes that were determined to be pregnant by transabdominal ultrasound and subsequently aborted or failed to raise lambs. However, toxoplasmosis can also have impacts on early pregnancy before pregnancy scanning, including embryonic death, resorption and early foetal mortality [29]. Therefore, it is possible that infections occurring early in pregnancy could result in primiparous ewes being not pregnant at scanning. This study did not determine association between *T. gondii* seropositivity and pregnancy status at scan 1. The low seropositivity reported in this study for primiparous ewes that were pregnant at scan 1 suggests that toxoplasmosis was unlikely to be an important contributor to early reproductive losses on these farms. Further investigation is required to determine if infection in early pregnancy is an important contributor to early pregnancy loss evident as ewes that are not pregnant at scanning.

*Toxoplasma gondii* seropositivity was detected for 57% of farms in this study, providing further evidence for the parasite’s endemicity and suggesting that exposure to *T. gon*dii occurs on many Australian sheep farms. This was consistent with other Australian studies reporting farm-level seroprevalence ranging 41–97% (Additional file 1). Seropositivity in mature ewes (8.1%) was within the 7–57% range reported for other Australian studies in the last 15 years, and similar to national seroprevalence 11.5% (46/401) for mutton (mature sheep) reported by Hamilton et al in a recent Australian abattoir survey (Additional file 1). Individual-animal *T. gondii* seropositivity for primiparous ewes in our study (1.1%) was lower than two previous studies reporting seroprevalence of 15 and 17% for Australian slaughter-age lambs in abattoir surveys (Additional file 1). Our study used a commercial indirect ELISA that has good sensitivity and specificity relative to latex agglutination test for sheep sera reported by the manufacturer [30], suggesting that differences in testing methodology are unlikely to explain the difference in seroprevalence between the studies. It is therefore more likely that the low seropositivity in primiparous ewes reflects the sporadic nature of *T. gondii* infection in Australian sheep rather than differences in serological methods.

Flock and animal seropositivity in mature ewes indicated variable exposure to *T. gondii* on Australian sheep farms. Most seropositive mature ewes were concentrated on five farms where within-farm seropositivity ranged 25–50%. Variable seropositivity in Australian sheep contrasts to the more consistent exposure reported for sheep farms in New Zealand, United States and United Kingdom [8, 31, 32], and suggests sporadic point source exposure to oocysts, likely via contaminated drinking water or feed source, are the major source of *T. gondii* exposure on Australian sheep farms. The higher (50%) seropositivity observed for ewes on a farm on Kangaroo Island (Additional file 3) was consistent with previous studies reporting high *T. gondii* seroprevalence on Kangaroo Island [10, 33]. Interestingly, there was no evidence of seropositivity in the primiparous ewes from the farm on Kangaroo Island, consistent with sporadic exposure to *T. gondii*. The risk of *T. gondii* exposure is associated with a range of factors including abundance of the feline definitive host, access to surface water and rainfall [34, 35]. Further work is warranted to determine regional differences in *T. gondii* exposure and incidence of toxoplasmosis. Improved understanding of regional variation and risk factors for *T. gondii* exposure on Australian sheep farms would inform cost-benefit analyses for interventions to reduce the risk of toxoplasmosis.

Sampling primiparous ewes was biased towards ewes that were determined to be pregnant and subsequently failed to rear a lamb. If toxoplasmosis was an important contributor to foetal and lamb mortality on these farms, then this sampling bias could result in overestimation of seroprevalence relative to the general population. Notwithstanding this, the low frequency of seropositivity and seroconversions during pregnancy in primiparous ewes that failed to raise lambs does not support the role of *T. gondii* as an important cause of reproductive losses between scanning and marking on these farms. A case control study would allow for determination of odds ratios for ‘fail to rear’ for ewes that were seropositive for *T. gondii*. However, due to the very low frequency of seropositivity to *T. gondii* in these flocks we therefore decided to target ‘fail to rear’ ewes to improve our confidence in the level of seropositivity in this cohort.

One ewe (Ewe 3479; Table 3) was seronegative at two timepoints after testing seropositive at scan 1 then subsequently tested seropositive at marking. The reason for fluctuating serostatus was not determined. However, this could be due to false positive results, issues with test reproducibility, or failure of the test to detect fluctuating titres below the detection limit. There is a lack of published data validating the test against other ‘gold standard’ tests such as microagglutination tests or PCR. A commercial modified agglutination test for *T. gondii* was validated for Australian sheep [36], but this test is no longer available. The indirect ELISA used in this study was found to have good agreement with modified agglutination test on a subset of sheep sera collected from another study (Additional file 5). Further validation of commercial indirect ELISA for natural *T. gondii* infections in Australian sheep will inform improved estimation of true prevalence for field studies.

Whilst the observations from this study do not support the need for widespread routine vaccination to reduce foetal and lamb mortality for primiparous ewes in Australia, *T. gondii* is endemic on Australian farms and associated with sporadic reproductive losses on some farms [9]. The low rates of seropositivity in primiparous and multiparous ewes suggests a lack of protective immunity in a large proportion of ewes [7]. These ewes remain susceptible to reproductive disease if a toxoplasmosis outbreak was to occur during gestation. Control of feral and domestic cat populations on sheep farms and measures to prevent contamination of feed and water sources from cat faeces can reduce the risk of toxoplasmosis outbreaks in susceptible sheep. Vaccination could be warranted in some regions where high farm and individual animal seroprevalence is identified [10], and high incidence of reproductive losses due to *T. gondii* are confirmed using foetal and lamb necropsy and laboratory investigation. The interpretation of *T. gondii* serology for the purpose of diagnosing toxoplasmosis is challenging, particularly for field investigations of reproductive loss in extensive sheep production systems where abortions or unusually high incidence of perinatal mortality are challenging to detect at the time losses are occurring. Diagnosis of toxoplasmosis should be supported with detection of *T. gondii* in tissues where possible, as well as the exclusion of other endemic pathogens.

## Conclusion

Toxoplasmosis was not a significant contributor to abortion and perinatal lamb mortality for primiparous ewes on farms across southern Australia. Seropositivity to *T. gondii* for mature multiparous ewes was detected by indirect ELISA on more than half of the farms included in this study. However, only 1% of primiparous ewes that had confirmed pregnancy and subsequently failed to raise a lamb had evidence of *T. gondii* seroconversion after mating. Low frequency of *T. gondii* seropositivity in primiparous ewes during gestation was consistent with the absence of detection of *T. gondii* using qPCR on aborted and stillborn lambs from a subset of farms.

## Methods and study design

### Study design, animals and research sites

**Fig. 1:**
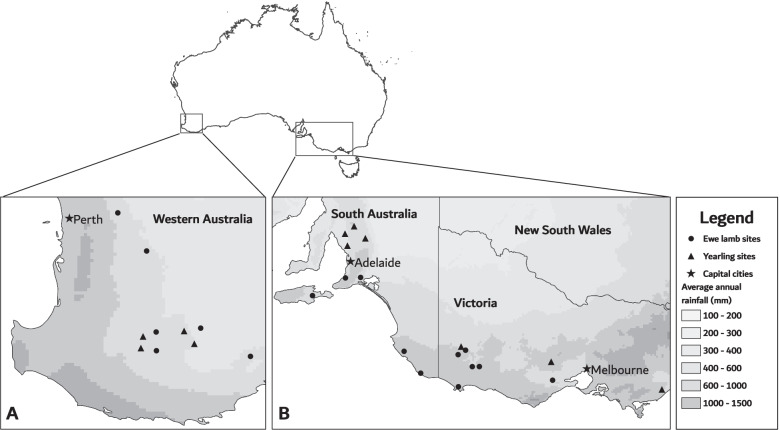
Approximate location of farms sampled in Western Australia (**A**) and South Australia and Victoria (**B**) adapted from Clune et al. [26]

This cohort study was conducted as part of a larger project investigating reproductive performance of primiparous using 30 primiparous ewe flocks on 28 farms located in Western Australia (*n =* 11), South Australia (*n =* 9), and Victoria (*n =* 10) (Fig. 1) between 2018 and 2020 as previously described [13]. Briefly, farms were located over a wide geographic area that incorporated different rainfall zones (Fig. 1 and Additional file 2). Farms were selected for inclusion based on; having at least 200 primiparous ewes available for the study, capacity to monitor ewes and their progeny over the study period, and with sheep genotype and management that were generally representative of standard commercial sheep farms in the region. Some farms included in the study managed flocks of stud sheep which may increase frequency of monitoring relative to commercial flocks, but the housing (*i.e.* all flocks were managed in paddocks for the duration of the study) and stocking intensity were broadly comparable to commercial sheep flocks in these regions.

Each flock was monitored during gestation and lambing. Merino and non-Merino breeds were included in the study (Additional file 2). Non-Merino flocks included Border Leicester, Dorper, White Suffolk and composite breeds. Ewes were mated as either ewe lambs (7–10 months, *n* = 19 flocks) or primiparous yearlings (18–20 months, *n* = 11 flocks). Most ewes were naturally mated, and all rams used were confirmed to be negative for *Brucella ovis* via serology prior to mating. At each farm, twenty mature multiparous ewes aged 3 years or older that had been bred on the same farm were randomly selected for blood sampling only (Additional file 3). All farms ran self-replacing flocks and ewes included in the study were managed according to standard farm practice.

### Animal measurements and sample collection

Monitoring of ewes and determination of pregnancy outcome are described by Clune et al. [13]. Briefly, foetal mortality for primiparous ewes was determined based on sequential transabdominal pregnancy ultrasounds (scans). Scan 1 was conducted approximately 85 days (range 62–101) from the start of mating. Scan 2 was conducted at least 30 days after the first scan at approximately 118 days (range 107–136) from the start of mating. The outcome of pregnancy and perinatal lamb mortality were determined based on number (single, twin or triplet) and survival status (lambs dead or alive) for lambs at lambing rounds and at lamb marking (approximately 6 weeks from the start of lambing). Ewe lactation status (lactating or not lactating) was determined by visual observation and/or palpation of the udder at lamb marking. Mid-pregnancy abortion was determined based on loss of pregnancy (i.e., no foetus/es detected) or foetal mortality (*i.e*., no evidence of foetal viability) between scan 1 and scan 2, and validated using lambing records (i.e. no lamb allocated to ewe at lambing rounds) and udder inspection (i.e. no evidence of lactation) at marking as described by Clune et al. [13]. Ewes that “failed to rear” were determined based on evidence of pregnancy at scan 1 with no live lamb present at lamb marking and no evidence of lactation at lamb marking.

Blood sampling of primiparous ewes was conducted as previously described [26, 27]. Briefly, blood samples for primiparous ewes were collected at five timepoints: pre-mating, scan 1, scan 2, pre-lambing (approximately 140 days from start of mating) and lamb marking (approximately 6 weeks from start of lambing). Blood samples for mature ewes were obtained at a single timepoint during the study period, but timing of sampling relative to lambing and their reproductive outcome was not recorded (Additional File 3). All blood samples were obtained by jugular venepuncture into serum vacutainer tubes with clot activator and stored on ice or at 2 °C. Within 72 h of collection, blood samples were centrifuged at 4000 rpm for 10 min and serum was decanted into 2 mL low protein-binding polypropylene screw cap micro tubes and stored at -20 °C prior to serological testing.

### Serology sample selection

The sample size necessary to estimate true prevalence was 239 ewes based on assumed true seroprevalence prevalence of 15% in primiparous ewes, assumed test sensitivity 90%, assumed specificity 99% and desired precision 5%. As 30 flocks were included in the study, this sample size was achieved with at least 8 ewes sampled per flock.

All samples from mature ewes and a sub-sample of at least 40 primiparous ewes from each flock were selected for *T. gondii* serology as previously described [26, 27]. Briefly, samples for primiparous ewes that were identified as pregnant at the first pregnancy scan but failed to successfully rear a lamb were prioritised for screening. That is, sample selection for primiparous ewes was biased towards ewes that were pregnant and failed to rear a lamb. This included primiparous ewes that were determined to have aborted and ewes for which lamb mortality occurred in the perinatal period. Samples for primiparous ewes that reared lambs were also included for screening where flocks had less than 40 ewes that failed to rear a lamb (Additional file 2). Blood samples collected at lamb marking were used for serology where available. For primiparous ewes where blood samples from marking were not available, blood samples collected at the latest available timepoint were used (i.e. blood sample collected at scan 2 or pre-lambing after foetal mortality was detected).

For primiparous ewes that returned a ‘positive’ result using indirect ELISA at the last time-point, serum samples from earlier timepoints (pre-mating, scan 1, scan 2 and pre-lambing) were tested to determine the timing of seroconversion relative to gestation and foetal or lamb mortality.

### Toxoplasma gondii serology

Anti-*T. gondii* IgG seropositivity was determined using commercial indirect ELISA (ID Screen Toxoplasmosis Indirect Multispecies, ID Vet, France) according to the manufacturer’s instructions [30]. Testing was performed by VETPATH Laboratories (Perth, Western Australia). The results were read at 450 nm using a Multiskan FC, Thermo Scientific spectrophotometer. Positive and negative internal controls were included with each plate. Optical density (OD) values were expressed as the mean percentage of sample/positive (S/*P*) values, as recommended by the manufacturer: S/*P* value = (OD_sample_ − OD_negative.control_)/(OD_positive control_ − OD_negative.control_). Serum samples were classified as positive (S/*P* value ≥50), doubtful (S/*P* value 40 to < 50) or seronegative (S/*P* value < 40) according to the manufacturer’s recommendation. This assay has a specificity of 100% (95% CI 98.2, 100) and a sensitivity of 100% (95% CI 89.1, 100) using sheep sera validated against latex agglutination test (MAST Group) according to the manufacturer’s internal validation report [30]. A subset of sera from Australian sheep previously tested with a validated modified agglutination test [36] in a separate study were re-tested using the commercial indirect ELISA, with sensitivity 90.5% (95% CI 71.1, 98.3) and specificity 100% (95% CI 64.6, 100) (Additional file 5).

Samples that returned a ‘doubtful’ result (Additional file 6) and a subset of samples that returned a negative result (Additional file 7) were re-tested using an alternate commercial ELISA test (IDEXX Toxotest, IDEXX Laboratories, Switzerland), according to manufacturer’s instructions. Re-testing was performed by Department of Primary Industry and Regional Development Diagnostic Laboratory Service. Each plate included positive and negative controls. The results were read at 450 nm using a Multiskan EX, Thermo Fisher Scientific spectrophotometer. Optical density (OD) values were expressed as the mean percentage of sample/positive (S/*P*) values, as recommended by the manufacturer: S/*P* value = (OD_sample_ − OD_negative.control_)/(OD_positive control_ − OD_negative.control_). Serum samples were classified as positive (S/*P* value ≥100), weak positive (S/*P* value 30 to < 100), suspect (S/*P* value 20 ≤ 30) or negative (S/*P* value < 20) according to the manufacturer’s recommendation. This assay has reported specificity 97.5% (95% CI 92.5, 99.4) and a sensitivity 90.9% (95% CI 83.4, 95.6) for sheep [37].

Samples that were ‘doubtful’ for the first test (using ID Screen®) but ‘positive’ or ‘weak positive’ for the second test (using IDEXX Toxotest) were categorised ‘positive’. Results that were ‘doubtful’ for first test and ‘negative’ or ‘suspect’ for second test were considered negative (Additional file 6).

### Statistical methods

Lamb mortality was calculated based on the number of lambs alive at marking expressed as a proportion of the number of foetuses identified at scan 1. Lamb mortality was classified as ‘mid-pregnancy abortion’ based on detection of pregnancy loss between scan 1 and scan 2 (and validated with lambing records and ewe lactation status). Mid-pregnancy abortion was expressed as a proportion (%) of ewes with abortion detected between scan 1 and scan 2 relative to the number of ewes that were confirmed pregnant at scan 1.

Apparent *T. gondii* seropositivity was calculated using the number of ewes categorised as positive expressed a proportion (%) of the ewes tested, with 95% confidence intervals determined using Jeffreys method [38]. Proportion *T. gondii* seropositivity were compared for the ewe age categories and states using a two-tailed two-sample proportion z-test.

The true seropositivity and 95% credible intervals (95% CrI) were estimated using Bayesian inference, considering the sensitivity and specificity and their 95% CrI derived from manufacturer’s internal validation report [30] as beta-pert distribution for priors [39].

## Supplementary Information

**Additional file 1.**

**Additional file 2.**

**Additional file 3.**

**Additional file 4.**

**Additional file 5.**

**Additional file 6.**

**Additional file 7.**

## Data Availability

The datasets generated and/or analysed during the current study not openly available due to privacy of participating enterprises, and are available from the corresponding author on reasonable request. Datasets generated are stored in a controlled access repository by Murdoch University.

## Abbreviations

CI: Confidence interval
CrI: Credible interval
ELISA: Enzyme-linked immunosorbent assay
qPCR: Quantitative polymerase chain reaction
