# Supplementary material for: Toxoplasma gondii is not an important contributor to poor reproductive performance of primiparous ewes from southern Australia: a prospective cohort study
Source: BMC Vet Res. 2022 Mar 19;18:109. doi: 10.1186/s12917-022-03211-w (PMC8933891; doi:10.1186/s12917-022-03211-w)
Supplement: Supplementary file 1 — Additional file 1. [file 12917_2022_3211_MOESM1_ESM.pdf]

Additional File 1

Individual animal and flock-level *T. gondii* seroprevalence values from seroprevalence surveys conducted across Australia between 1975 and 2021.

Study	State(s)^	Individual animal seroprevalence *n* (%)	Flock-level seroprevalence *n* (%)
Munday [1]	TAS	Lambs: 27/160 (16.9%)<br>Adults: 89/144 (61.7%)	
O'Donoghue et al. [2]	SA	86/ 1159 (7.4%)	27/59 (45.8%)
Plant et al. [3]	NSW	515/5724 (9%)	219/534 (41%)
Kiermeier et al. [4]	NSW, VIC, QLD, SA, WA	Lambs: 37/246 (14.9%)<br>Adults: 126/388 (32.5%)	
McGregor and Harvey [5]	NSW	33/489 (6.7%)	6/10 (60%)
Taggart et al. [6]	SA	318/560 (57%)	
Lanyon and O'Handley [7]	SA	209/875 (23.9%)	28/29 (96.6%)
Hamilton et al. [8]		46/401 (11.5%)	

^NSW: New South Wales; QLD: Queensland; SA: South Australia; TAS: Tasmania; WA: Western Australia; VIC: Victoria.

References cited:

1. Munday BL: **Prevalence of toxoplasmosis in Tasmanian meat animals**. *Aust Vet J* 1975, **51**(6):315-316.
2. O'Donoghue PJ, Riley MJ, Clarke JF: **Serological survey for *Toxoplasma* infections in sheep**. *Aust Vet J* 1987, **64**(2):40-45.
3. Plant JW, Freeman P, Saunders E: **Serological survey of the prevalence of *Toxoplasma gondii* antibodies in rams in sheep flocks in New South Wales**. *Aust Vet J* 1982, **59**(3):87-89.
4. Kiermeier A, Hamilton D, Smith G: **National serological baseline survey of Toxoplasma gondii in lambs and sheep**. Australia MaL; North Sydney: 2008: Available at: Accessed:
5. McGregor H, Harvey R: **A pilot survey of the prevalence of serum antibodies against *Toxoplasma gondii* and risk factors for transmission of *T. gondii* to sheep in the Tumbarumba shire of New South Wales** Flock & Herd; 2011. Available at: [http://www.flockandherd.net.au/sheep/reader/toxoplasmosis.html] Accessed: May 2018
6. Taggart P, McAllister M, Rutley D, Caraguel C: **Oesophageal sarcocystosis observed at slaughter provides a reliable and efficient proximate measure of *Toxoplasma gondii* seroprevalence in sheep**. *Aust Vet J* 2020, **98**(7):305-311.
7. Lanyon SR, O'Handley RM: **Relationship between *Toxoplasma gondii* seroprevalence and lamb marking in South Australian sheep flocks**. *Aust Vet J* 2020, **98**(11):525-528.
8. Hamilton D, Hodgson K, Howard A, Jolley J, Mahbub K, Torok V, McAllister M: **Investigation of the viability and national serological prevalence of *Toxoplasma gondii* in Australian sheep (Final Report V.MFS.0419)**. Meat and Livestock Australia; North Sydney, Australia: 2021: Available at: https://www.mla.com.au/research-and-development/reports/2019/toxoplasma-gondii-sheep/# Accessed: September 2021
