# Supplementary material for: Toxoplasma gondii is not an important contributor to poor reproductive performance of primiparous ewes from southern Australia: a prospective cohort study
Source: BMC Vet Res. 2022 Mar 19;18:109. doi: 10.1186/s12917-022-03211-w (PMC8933891; doi:10.1186/s12917-022-03211-w)
Supplement: Supplementary file 2 — Additional file 2. [file 12917_2022_3211_MOESM2_ESM.pdf]

Additional File 2

Apparent *T. gondii* seropositivity with 95% confidence interval (95% CI) in primiparous ewes from Australian farms determined using indirect ELISA.

			Tested (*n*)			***T. gondii* serology**
**Flock reference**	**Location**	**Breed**	Aborted or FTR[C]	Reared lamb	Total	Seropositive *n* (%)
**YEARLINGS[A]**						
1	Kojonup, WA	Merino	40	0	40	0
2	Kojonup, WA	Merino	40	0	40	0
5	Korunya, SA	Merino	40	0	40	0
6	Bagot Well, SA	Merino	NA	NA	40*	1 (2.5%)
9	Watervale, SA	Merino	30	10	40	0
10	Broomehill, WA	Merino	40	0	40	2 (5%)
12	Tarlee, SA	Merino	40	0	40	1 (2.5%)
13	Giffard West, VIC	Merino	40	0	40	1 (2.5%)
15	Katanning, WA	Merino	40	0	40	1 (2.5)
26	Culla, VIC	Merino	40	0	40	0
29	Ballarat, VIC	Merino	30	10	40	1 (2.5%)
**Seroprevalence** % (95% CI)			380	20	440	1.59% (0.7, 3.1)
**EWE LAMBS[B]**						
3 [d]	Narrogin, WA	Non-Merino	50	48	98	1 (1%)
4	York, WA	Non-Merino	31	9	40	0
7	Kojonup, WA	Non-Merino	26	14	40	1 (2.5%)
8	Katanning, WA	Merino	30	10	40	0
11	Kojonup, WA	Non-Merino	35	5	40	0
14 [d]	Narrogin, WA	Non-Merino	40	0	40	0
16	Ongerup, WA	Non-Merino	40	0	40	0
17	Hamilton, VIC	Non-Merino	20	20	40	0
18	Hamilton, VIC	Non-Merino	39	1	40	0
19 [e]	Nareen, VIC	Non-Merino	40	0	40	0
20	Cashmore, VIC	Non-Merino	40	0	40	2 (5%)
21	Coojar, VIC	Non-Merino	40	0	40	1 (2.5%)
22	Mount Gambier, SA	Non-Merino	40	0	40	1 (2.5%)
23	Kangaroo Island, SA	Non-Merino	29	11	40	0
24	Beachport, SA	Non-Merino	61	0	61	1 (1.6%)
25	Sellicks Hill, SA	Non-Merino	40	0	40	0
27 [e]	Nareen, VIC	Non-Merino	37	3	40	0
28	Inverleigh, VIC	Non-Merino	39	1	40	0
30	Strathalbyn, SA	Non-Merino	40	0	40	0
**Seropositivity** % (95% CI)			717	122	839	0.83% (0.37, 1.63)
**Overall seropositivity** (% (95% CI))			1097	142	1279	1.09% (0.63, 1.78)

[a] Primiparous ewes mated at approximately 18 months of age
[b] Primiparous ewes mated at approximately 8 months of age
[c] FTR: fail to rear (scanned pregnant with subsequent abortion or perinatal lamb mortality)
[d] Same property with Flock 3 sampled 2018 and Flock 14 sampled 2019
[e] Same property with Flock 19 sampled 2019 and Flock 27 sampled 2020
WA: Western Australia SA: South Australia VIC: Victoria
*Reproductive outcome unknown
