# Supplementary material for: Toxoplasma gondii is not an important contributor to poor reproductive performance of primiparous ewes from southern Australia: a prospective cohort study
Source: BMC Vet Res. 2022 Mar 19;18:109. doi: 10.1186/s12917-022-03211-w (PMC8933891; doi:10.1186/s12917-022-03211-w)
Supplement: Supplementary file 3 — Additional file 3. [file 12917_2022_3211_MOESM3_ESM.pdf]

Additional File 3

Apparent *T. gondii* seropositivity for multiparous mature ewes (3 years of age or older) from Australian farms determined using indirect ELISA.

Flock reference	Location	Age	*T. gondii* serology Tested *n*	Seropositive *n* (%)
1	Kojonup, WA	4-7years	20	0
2	Kojonup, WA	5 years	20	0
3 and 14 [a]	Narrogin, WA	5 years	20	2 (10%)
4	York, WA	5-6 years	20	0
5	Korunya, SA	5+ years	20	0
6	Bagot Well, SA	5+ years	20	0
7	Kojonup, WA	5-7 years	20	2 (10%)
8	Katanning, WA	4-6 years	20	0
9	Watervale, SA	5+ years	18	0
10	Broomehill, WA	5-10 years	20	0
11	Kojonup, WA	5-6 years	20	0
12	Tarlee, SA	5+ years	20	0
13	Giffard West, VIC	5 years	20	0
15	Katanning, WA	5-7 years	20	1 (5%)
16	Ongerup, WA	4-7 years	20	0
17	Hamilton, VIC	4-5 years	20	0
18	Hamilton, VIC	6-7 years	20	1 (5%)
19 and 27 [b]	Nareen, VIC	5-8 years	20	0
20	Cashmore, VIC	4-8 years	20	7 (35%)
21	Coojar, VIC	4+ years	20	0
22	Mount Gambier, SA	3-9 years	20	1 (5%)
23	Kangaroo Island, SA	4-7 years	20	10 (50%)
24	Beachport, SA	5+ years	20	1 (5%)
25	Sellicks Hill, SA	4-7 years	20	5 (25%)
26	Culla, VIC	4-9 years	20	0
28	Inverleigh, VIC	3-6 years	20	6 (30%)
29	Ballarat, VIC	4-9 years	20	9 (45%)
30	Strathalbyn, SA	5+ years	20	0
**Seropositivity** (% (95% confidence interval)			558	8.06% (6.02, 10.54)

[a] Primiparous ewes from property sampled in 2018 (flock 3) & 2019 (flock 14) – mature ewes sampled in one year only

[b] Primiparous ewes from property sampled in 2019 (flock 19) & 2020 (flock 27) – mature ewes sampled in one year only

WA: Western Australia SA: South Australia VIC: Victoria
