# Supplementary material for: Toxoplasma gondii is not an important contributor to poor reproductive performance of primiparous ewes from southern Australia: a prospective cohort study
Source: BMC Vet Res. 2022 Mar 19;18:109. doi: 10.1186/s12917-022-03211-w (PMC8933891; doi:10.1186/s12917-022-03211-w)
Supplement: Supplementary file 4 — Additional file 4. [file 12917_2022_3211_MOESM4_ESM.pdf]

Additional File 4: Molecular detection of *Toxoplasma gondii* in tissues and histopathology for aborted and stillborn lambs reported by Clune et al. [1]

Methods

Tissue samples from aborted (*n* = 2) or stillborn (*n* = 33) lambs recovered from a subset of seven flocks of primiparous ewes (Flocks 1, 2, 3, 7, 11, 14, 16) from six farms in Western Australia were submitted to the Department of Primary Industry and Regional Development Diagnostic Laboratory Services (Perth, Western Australia).

Tissue samples were screened for *T. gondii* using qPCR as previously described [1]. Briefly, DNA extraction was performed using the QIAamp DNA Mini Kit (Qiagen) and run on the automated Qiacube platform (Qiagen) following the protocol for purification of DNA from tissues and the qPCR conducted using VetMAX™ *T. gondii* Kit (Thermo Fisher Scientific) according to manufacturer instruction.

Histopathology was performed on formalin fixed tissues processed to haematoxylin and eosin (H&E) slides; a subset of cases was also subjected to immunohistochemistry. Representative specimens were processed from 10% buffered formalin solution to paraffin embedded tissue in a Logos Milestone histological processer and blocked using standard histological techniques. Sections were trimmed at 4µm thickness and stained to H&E in a Leica autostainer XL with Leica CV5030 coverslipper. Selected sections were subject to immunohistochemistry with an anti-*Chlamydia* polyclonal antibody (B47829R, Progen) and an anti-*Toxoplasma gondii* polyclonal antibody (B65201R, Biodesign). Both antibodies were visualised using the Dako Envision Dual-link system and Dakocytomation DAB+ (both Dako, Agilent) according to the manufacturers' instructions.

Results

Toxoplasma gondii was not detected in any tissue samples from 35 aborted or stillborn lambs recovered from primiparous ewes from seven flocks on the subset of six farms in Western Australia [1].

One aborted lamb recovered from a primiparous ewe lamb flock in Victoria (Flock 19) between scan 2 and pre-lambing had evidence of multifocal encephalitis suggestive of protozoal disease. However, no protozoal organisms were observed upon histological examination, and there was no evidence of *T. gondii* seropositivity for primiparous ewes in this flock (Additional file 1). There was also no evidence of *T. gondii* seropositivity for primiparous ewes from the same property sampled in the following year (Flock 27; Additional file 1), nor mature ewes on the same property (Additional file 2). *Campylobacter fetus* was cultured from the same aborted foetus and other aborted foetuses from the same flock, indicating that campylobacteriosis was the most likely cause of abortion in this flock.

References cited

1. Clune T, Besier S, Hair S, Hancock S, Lockwood A, Thompson A, Jelocnik M, Jacobson C: ***Chlamydia pecorum* detection in aborted and stillborn lambs from Western Australia**. *Vet Res* 2021, **52**(1):84.
