# Supplementary material for: Toxoplasma gondii is not an important contributor to poor reproductive performance of primiparous ewes from southern Australia: a prospective cohort study
Source: BMC Vet Res. 2022 Mar 19;18:109. doi: 10.1186/s12917-022-03211-w (PMC8933891; doi:10.1186/s12917-022-03211-w)
Supplement: Supplementary file 5 — Additional file 5. [file 12917_2022_3211_MOESM5_ESM.pdf]

Additional File 5

Validation of *T. gondii* indirect ELISA (ID Screen Toxoplasmosis Indirect Multi-species, IDvet) with modified agglutination test

The indirect ELISA (ID Screen Toxoplasmosis Indirect Multispecies, ID Vet, France) was validated in-house against a panel of 28 Australian sheep serum previously tested using the modified agglutination test (Toxo-Screen Direct Agglutination kit, BioMérieux, Marcy-l'Étoile France) [1]. Samples were analysed according to the manufacturer's instructions for both tests.

Indirect ELISA (ID Screen)	Value (%)	95% confidence interval
Sensitivity	90.5	71.1, 98.3
Specificity	100	64.6, 100
Positive Predictive Value	100	83.2, 100
Negative Predictive Value	77.8	45.3, 96

References cited:

1. Hamilton D, Hodgson K, Howard A, Jolley J, Mahbub K, Torok V, McAllister M: **Investigation of the viability and national serological prevalence of *Toxoplasma gondii* in Australian sheep (Final Report V.MFS.0419)**. Meat and Livestock Australia; North Sydney, Australia: 2021: Available at: https://www.mla.com.au/research-and-development/reports/2019/toxoplasma-gondii-sheep/# Accessed: September 2021
