# Supplementary material for: Toxoplasma gondii is not an important contributor to poor reproductive performance of primiparous ewes from southern Australia: a prospective cohort study
Source: BMC Vet Res. 2022 Mar 19;18:109. doi: 10.1186/s12917-022-03211-w (PMC8933891; doi:10.1186/s12917-022-03211-w)
Supplement: Supplementary file 6 — Additional file 6. [file 12917_2022_3211_MOESM6_ESM.pdf]

Additional File 6

Serological status category for 'doubtful' samples re-tested for anti-*T. gondii* IgG with alternate indirect ELISA

Sample ID	Flock reference	Reproductive outcome	ID-VET ELISA Result (S/P value [a])	IDEXX ELISA Result (S/P value [a])	Serological category
**Primiparous ewes**					
3311	2	Perinatal loss	Doubtful (40.71)	Negative (6.24)	Negative
3387	2	Perinatal loss	Doubtful (41.50)	Negative (11.84)	Negative
3548	3	Perinatal loss	Doubtful (40.88)	Negative (5.21)	Negative
2240*	3	Late abortion/ perinatal loss	Doubtful (46.51)	Negative (3.90)	Negative
2804*	3	Late abortion/ perinatal loss	Doubtful (49.93)	Negative (9.41)	Negative
15115	6	Unknown	Doubtful (41.45)	Negative (3.56)	Negative
16381	7	Late abortion/ perinatal loss	Doubtful (45.92)	Negative (3.95)	Negative
16474	8	Raised twins	Doubtful (43.43)	Negative (4.24)	Negative
16476	8	Perinatal loss	Doubtful (42.84)	Negative (5.90)	Negative
16827	8	Raised single	Doubtful (48.99)	Negative (11.74)	Negative
17797	12	Perinatal loss	Doubtful (41.77)	Negative (3.56)	Negative
17870	12	Perinatal loss	Doubtful (46.29)	Negative (3.02)	Negative
11358*	15	Perinatal loss	Doubtful (43.21)	Negative (2.19)	Negative
15681*	15	Perinatal loss	Doubtful (42.55)	Negative (2.68)	Negative
18306	17	Perinatal loss	Doubtful (40.59)	Negative (3.17)	Negative
7302*	20	Perinatal loss	Doubtful (43.95)	Negative (13.60)	Negative
12499*	20	Perinatal loss	Doubtful (45.61)	Weak positive (33.67)	Positive
16178*	20	Perinatal loss	Doubtful (46.48)	Weak positive (70.52)	Positive
17987	21	Perinatal loss	Doubtful (41.26)	Negative (4.58)	Negative
7688*	21	Perinatal loss	Doubtful (43.54)	Negative (8.09)	Negative
18201	22	Perinatal loss	Doubtful (46.08)	Negative (5.95)	Negative
21895	24	Aborted	Doubtful (47.50)	Negative 0.88)	Negative
**Mature ewes**					
M140	11	Unknown	Doubtful (49.84)	Negative (4.24)	Negative
M549	22	Unknown	Doubtful (46.34)	Negative (2.92)	Negative
M552	22	Unknown	Doubtful (40.45)	Negative (2.58)	Negative
M554	22	Unknown	Doubtful (43.92)	Negative (2.10)	Negative
M555	22	Unknown	Doubtful (47.51)	Negative (4.87)	Negative
M288	24	Unknown	Doubtful (46.71)	Negative (9.84)	Negative
M304	25	Unknown	Doubtful (40.86)	Negative (0.97)	Negative
M308	25	Unknown	Doubtful (46.09)	Negative (1.95)	Negative
M466	26	Unknown	Doubtful (47.23)	Negative (2.92)	Negative
M525	29	Unknown	Doubtful (44.63)	Negative (14.33)	Negative
M534	29	Unknown	Doubtful (45.61)	Negative (12.33)	Negative

[a] S/P value: mean percentage of sample/positive $((OD_{sample} - OD_{negative.control})/(OD_{positive\ control} - OD_{negative.control}))$

* Serial blood sample from timepoint prior to lamb marking tested to determine timing of seroconversion
