# Supplementary material for: Toxoplasma gondii is not an important contributor to poor reproductive performance of primiparous ewes from southern Australia: a prospective cohort study
Source: BMC Vet Res. 2022 Mar 19;18:109. doi: 10.1186/s12917-022-03211-w (PMC8933891; doi:10.1186/s12917-022-03211-w)
Supplement: Supplementary file 7 — Additional file 7. [file 12917_2022_3211_MOESM7_ESM.pdf]

Additional File 7

Serological status category for subset of ‘negative’ samples (ID Screen Toxoplasmosis Indirect Multi-species, IDvet) re-tested for anti-*T. gondii* IgG with alternate indirect ELISA (IDEXX Toxotest, IDEXX)

**Sample ID**	**Flock reference**	**Reproductive outcome**	**ID-VET ELISA** Result	**IDEXX ELISA** Result
3056	1	Perinatal loss	Negative	Negative
3057	1	Perinatal loss	Negative	Negative
3067	1	Perinatal loss	Negative	Negative
3108	1	Perinatal death + ewe death	Negative	Negative
3111	1	Late abortion/ perinatal loss	Negative	Negative
3347	2	Perinatal loss	Negative	Negative
3348	2	Perinatal loss	Negative	Negative
3350	2	Perinatal loss	Negative	Negative
3351	2	Perinatal loss	Negative	Negative
3358	2	Perinatal loss	Negative	Negative
3426	3	Not pregnant at scanning	Negative	Negative
3427	3	Reared lamb	Negative	Negative
3429	3	Abortion	Negative	Negative
3431	3	Perinatal loss	Negative	Negative
3432	3	Suspect perinatal loss	Negative	Negative
2964	4	Perinatal loss	Negative	Negative
2995	4	Perinatal loss	Negative	Negative
2998	4	Perinatal loss	Negative	Negative
3605	4	Perinatal loss	Negative	Negative
3614	4	Perinatal loss	Negative	Negative
